# Evaluation of Selected Properties of Ti Coatings Deposited on MED610 for Medical Applications

**DOI:** 10.3390/ma19010060

**Published:** 2025-12-23

**Authors:** Katarzyna Piotrowska, Joanna Kowalczyk, Tomasz Kozior

**Affiliations:** Faculty of Mechatronics and Mechanical Engineering, Kielce University of Technology, 25-314 Kielce, Poland; kpawelec@tu.kielce.pl (K.P.); jkowalczyk@tu.kielce.pl (J.K.)

**Keywords:** 3D printing, MED610, PJM, wettability, friction, wear

## Abstract

The aim of the study was to develop and evaluate the properties of MED610 material coated with a thin titanium (Ti) layer deposited by physical vapour deposition (PVD) for medical applications. The coating was designed to improve biocompatibility and selected functional characteristics, such as the material’s tribological resistance. Two groups of samples—unpolished and polished—were prepared to enable an assessment of the influence of surface topography on functional performance. A titanium layer was applied to both groups, followed by an analysis of the surface geometric structure, contact angle, and tribological properties in an artificial saliva environment with neutral pH, simulating oral cavity conditions. The results of these investigations allowed for a comprehensive assessment of the influence of surface topography and the presence of a Ti coating on the functional properties of MED610, a material approved for contact with soft tissues. The findings confirmed that the application of a titanium coating favourably affected the structure and durability of the material—the coating reduced surface roughness by 20–45% and exhibited good adhesion to the substrate. The polishing and coating processes significantly altered the tribological properties: they increased the coefficient of friction by approximately 31% while simultaneously reducing volumetric wear by up to 75% for uncoated samples and by 44% for samples with a Ti coating.

## 1. Introduction

The development of 3D printing technologies represents one of the key directions of modern manufacturing engineering, enabling the fabrication of components with complex geometries that are unattainable using conventional production methods. As knowledge of 3D printing technologies continues to expand, the number of their practical applications is also steadily increasing [1].

Additive manufacturing is currently used, among others, in the automotive and aerospace industries [2,3], as well as in medicine [4,5], where its design flexibility enables applications across numerous specialties, including surgery, dentistry, and orthopaedics. Such products include both ready-to-use components and highly personalised anatomical models, such as implants [6,7], surgical guides [8,9], preoperative simulations [7,10], biodegradable screws [11,12], functional prostheses and orthoses [1], as well as drug-delivery systems [13]. Moreover, MED610 can be used as an acrylic substitute in the production of replacement inserts for ocular plaque brachytherapy applicators [14], as well as for personalised nasoalveolar moulding devices designed for preoperative unilateral and bilateral cleft lip and palate treatment in infants [15]. It is also employed in the fabrication of customised mouthpieces for patients undergoing head and neck radiotherapy [16]. Consequently, intensive research is being conducted on new materials [4] that are characterised by biocompatibility [1,4] and good printability [1].

An example of a polymer used in medical applications is MED610. It is a biocompatible photopolymer employed in the PolyJet technology [17,18] used, among others, in dental applications. This material is approved for long-term skin contact—exceeding 30 days [18]—and for short-term contact with mucous membranes—up to 24 h [17,18]. MED610, obtained through the photopolymerisation of liquid polymer resins, was investigated by the authors of study [4]. The samples were manufactured in a high-accuracy mode with a layer thickness of 0.016 mm and three printing orientations in the X–Z plane: 0°, 45°, and 90°. The authors conducted tribological tests under technically dry friction conditions and with lubrication using a physiological saline solution (0.9% NaCl) at a load of 10 N. The counter samples were 6 mm diameter balls made of polyamide 6.6 and polyoxymethylene. The analysis of the results demonstrated that the printing orientation affects tribological wear, which is associated with the anisotropic nature of 3D printing technology. The lowest average coefficient of friction was obtained for samples printed in the 90° orientation.

In turn, the authors of study [18] conducted tribological tests on MED610 samples manufactured using PolyJet Modeling (PJM) technology. The tests were performed at three different loads (30, 45, and 60 N) and sliding speeds of 100, 150, and 200 m/s. Additionally, a metrological analysis was carried out to assess both the reliability of the printing technology and the material itself. The results showed that MED610 exhibits relatively good wear resistance. It was observed that the maximum wear depth and the wear track area decrease with increasing rotational speed. Nevertheless, this material is not suitable for use in friction nodes subjected to high loads. In turn, in the publication [19], the authors conducted tribological tests under rotary motion using samples made of M390 and M398 steels produced by powder metallurgy combined with HIP. These samples differed in surface roughness as well as carbide content—20% for M390 and 30% for M398. The counter sample was a 6.35 mm diameter Al_2_O_3_ ball. The tests were performed at a temperature of 200 °C under technically dry friction conditions. The authors demonstrated that M398 can replace M390, as it exhibits superior tribological properties. The M398 material showed more than a 400% reduction in wear compared with M390, along with a lower average friction coefficient by 0.05. However, in the publication [20], the authors investigated the effect of increasing irradiation doses of accelerated electrons on changes in the friction coefficient and surface roughness of the polymer materials PET, PTFE, and PE2000C. The materials were irradiated with three doses: 33 kGy, 129 kGy, and 300 kGy. Tribological tests were then carried out under reciprocating motion in technically dry friction conditions, with a G40 steel ball used as the counter sample. The applied load increased from 10 N to 100 N. Irradiation altered both the roughness and friction coefficient of the examined polymers: PET became smoother, whereas PE2000C became rougher. Higher doses—particularly 300 kGy—led to surface degradation and an increase in friction.

Materials used in medical applications, in addition to meeting specific mechanical and tribological requirements, must also exhibit non-toxicity. It is well known that uncured liquid photopolymer resins used in 3D printing processes display cytotoxic effects. They may cause severe eye irritation and allergic skin reactions upon prolonged contact. Only after the curing process do these materials become chemically stable and safe in their solid state. For this reason, all uncured residues must be removed during the cleaning stage of printed parts. However, depending on the printing orientation, uncured material may remain trapped within pores, channels, or internal cavities of the structure, which may lead to cytotoxicity during the final use of the product [1]. An effective way to reduce the toxic effects of uncured material residues is the application of thin, biocompatible protective coatings [21]. Such layers act as a barrier between the printed surface and the body’s tissues, ensuring the safe use of the medical device. An example of a material used for this purpose is titanium, which—due to its high strength-to-weight ratio and excellent biocompatibility [22]—is widely employed in implantology. This metal rarely induces allergic reactions, which is particularly important for components intended for long-term contact with the human body [23]. Moreover, titanium is generally regarded as non-toxic [23,24]—as it does not release harmful substances into the body, thereby ensuring patient safety over extended periods [23]. Consequently, titanium can be used in a wide range of medical applications, including surgical instruments, implants, and dental components [24]. According to Rodrigues et al. [25], a titanium (Ti) coating deposited on ultra-high molecular weight polyethylene (UHMWPE) exhibits superior mechanical properties—such as higher hardness and improved resistance to nanoscratching—as well as better tribological performance compared with tantalum (Ta) and zirconium (Zr) coatings. Among the analysed coatings, the Ti layer demonstrated the lowest coefficient of friction.

The authors of study [26], investigated metal coatings deposited on the polymer materials PEEK, UHMWPE, and PTFE, as well as the adhesion of osteoblasts to orthopaedic polymers coated with titanium or gold using the ion-plasma deposition process. As a result of this process, a nanostructured, surface-modified layer (with features below 100 nm) was obtained. It was reported that polymers coated with titanium or gold exhibited a significant increase in osteoblast adhesion compared with uncoated polymers. However, in study [27], the authors developed a new polymer coating process based on titanium-derived layers deposited using the PACVD method. Their findings indicated that thin and very smooth layers exhibit excellent adhesion to PET, PES, PTFE, and PVC polymers. For polyolefins such as PP and PE, the adhesion is lower, although it can be improved through preliminary plasma treatment. The researchers demonstrated that the coating enhances the biocompatibility and blood compatibility of the polymers. One possible factor contributing to improved biocompatibility may be the increased critical surface tension of the coating compared with uncoated polymers. Furthermore, the coating exhibited barrier properties, preventing the leaching of plasticisers—particularly from PVC—which is a widely discussed issue in the context of using PVC in medical applications.

In summary, despite the wide use of 3D printing technologies in medicine, photopolymer materials such as MED610 still require modification to improve their biocompatibility and wear resistance, as well as extremely precise and complex 3D printing procedures to eliminate toxicity associated with the printing process itself. Therefore, this study proposes an innovative solution involving the deposition of a thin titanium coating on MED610 prints, which also greatly facilitates the sterilisation of such models. The application of this modification aims to enhance the biological safety of the material, minimise the influence of uncured polymer residues, and improve its tribological properties under conditions simulating the oral environment.

## 2. Materials and Methods

### 2.1. Research Methodology

**Figure 1:**
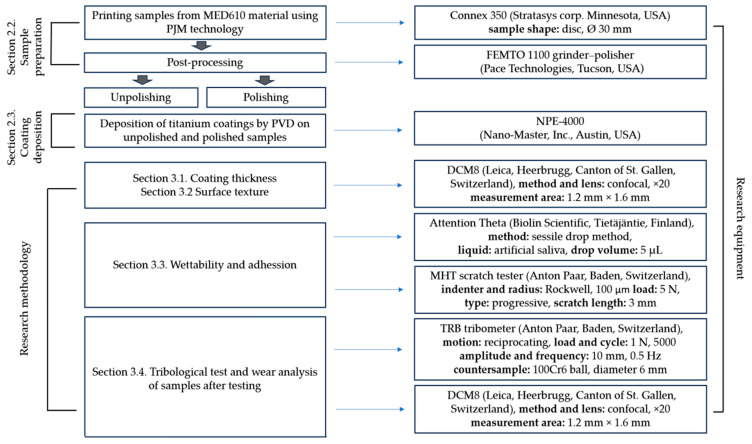
Research scheme and equipment used.

Figure 1 presents the research scheme and the equipment used, summarizing all relevant information on the performed tests and the devices on which they were conducted.

**Figure 2:**
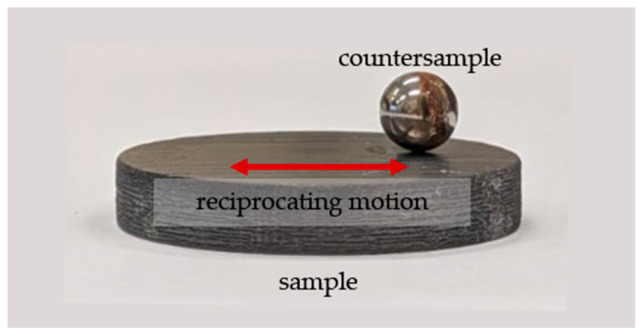
View of the friction pair.

Tribological tests were carried out in a classic ball-on-disc system with reciprocating motion at ambient temperature, with the artificial saliva solution being applied with a pipette to the sample surface and maintained at a constant volume throughout the test. The chemical composition of the lubricant is presented in Table 1 and a photograph of the friction node is presented in Figure 2.

### 2.2. Sample Preparation

The test samples were manufactured using the PolyJet Matrix technology, which involves the photo-curing of liquid polymer resins. In this technology, fine droplets of liquid resin are jetted onto the surface of the currently formed layer of the model, corresponding to its cross-sectional geometry, as well as onto the regions requiring support material. The samples were fabricated using a Connex 350 printer. They were designed as discs with a diameter of 30 mm and a height of 6 mm to facilitate both the coating deposition process and subsequent wear analysis.

The 3D printing process was carried out using predefined technological parameters, including a layer thickness of 0.016 mm, the high-quality printing mode, and the matt printing type. The matt setting ensured complete coverage of every surface of the sample with support material, enabling the production of a uniform surface across the entire specimen. After printing, the samples were initially cleaned of support material using a water under heigh pressure. According to the manufacturer’s instructions, the PJM technology is characterised by a printing resolution of 600 dpi in the X-axis, 600 dpi in the Y-axis, and 1600 dpi in the Z-axis. Furthermore, as demonstrated in the authors’ previous studies, the manufacturing accuracy in the X–Y plane is typically on the order of 50 micrometres. In the Z-axis, this value is generally lower due to the very small layer thickness of 15 micrometres.

All samples were printed in a single orientation on the build platform—the specimen was positioned with its flat frontal surface placed directly on the platform. This approach allowed for highly precise reproduction of the cylindrical surface.

MED610 is a material with very good medical performance characteristics. According to the product specification, the manufacturer states that the material has been developed for medical and dental applications [29]. Moreover, this resin meets the normative requirements for:

- irritation and type IV hypersensitivity [30];
- cytotoxicity [31];
- genotoxicity [32];
- chemical characterisation [33].

According to the material manufacturer and the Material Safety Data Sheet [34] the key components of MED610 include:

- Isobornyl acrylate, 15–30 wt %;
- Acrylic monomer, 15–30 wt %;
- Urethane acrylate, 10–30 wt %;
- Acrylic monomer, 5–15 wt %;
- Epoxy acrylate, 5–15 wt %;
- Acrylate oligomer, 5–15 wt %;
- Photoinitiator, 0.1–2 wt %.

**Figure 3:**
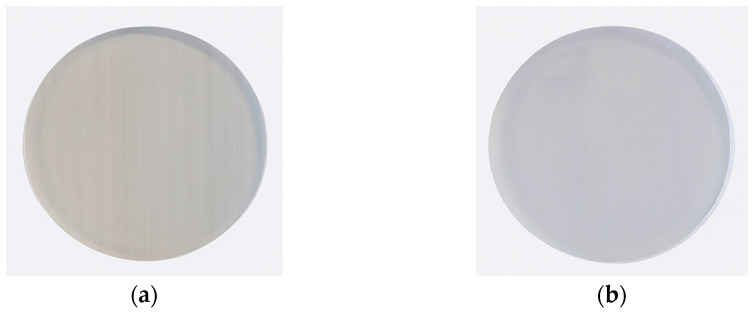
Photographs of samples before coating deposition: unpolished (**a**), polished (**b**).

Surface preparation constituted a crucial stage prior to coating deposition. A portion of the samples was subjected to grinding, while the remaining specimens were left in their as-printed state. Grinding was performed using silicon carbide abrasive papers with progressively increasing grit sizes—from 120 to 600. The coatings were applied both to the samples after initial cleaning following 3D printing (Figure 3a) and to the polished samples (Figure 3b). The surfaces prepared in this manner provided an appropriate substrate for the subsequent deposition of the titanium coating using the PVD method.

### 2.3. Coating Deposition

**Figure 4:**
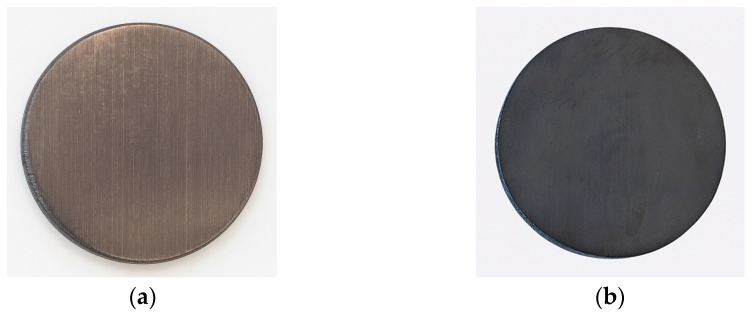
Photographs of samples with Ti coating: unpolished (**a**), polished (**b**).

The titanium coating was deposited using the physical vapour deposition (PVD) method with a Nanomaster system. Prior to initiating the process, the samples were thoroughly cleaned and subsequently placed inside the deposition chamber. In the first stage, cleaning was carried out in an argon (Ar) atmosphere under vacuum conditions for 20 min. This was followed by the deposition stage, performed using a titanium target. The coating process lasted 2 h. Detailed parameters for both the cleaning stage and the coating deposition are summarised in Table 2. Photographs of the coated samples are presented in Figure 4. As shown in Figure 4a, certain imperfections of the process are clearly visible, including traces resulting from the spreading of material by individual printing heads, whereas in Figure 4b—after polishing—all such irregularities have been removed.

## 3. Results

### 3.1. Coatings Thickness

**Figure 5:**
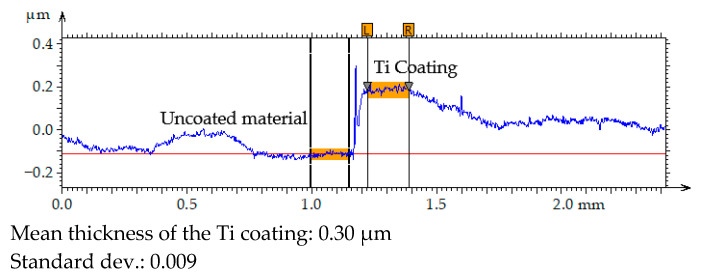
Primary profile of the surface texture–coating/substrate transition area.

The thickness of the Ti coating was measured using optical microscopy. To ensure accurate determination of the coating step height, the sample surface was mechanically polished prior to deposition, achieving a surface roughness of approximately Sa ≈ 100 nm. This preparation was necessary because measurements performed on unpolished surfaces are unreliable due to high topographical variability. After coating deposition, the thickness was determined by analysing the height difference (step height) between the coated and uncoated regions of the surface profile. The measurement was repeated five times at different locations to assess the uniformity of the coating. The results are presented in Figure 5. The mean thickness and standard deviation were calculated to evaluate the reproducibility of the deposition process.

The results of the coating thickness measurements indicated that the 2 h PVD process produced a layer with a thickness of approximately 300 nm.

### 3.2. Surface Texture

**Figure 6:**
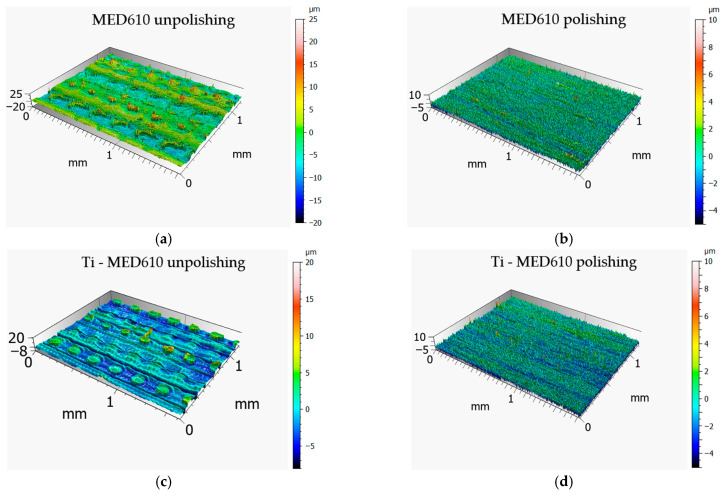
Surface topography of MED610 (**a**,**b**) and Ti–MED610 (**c**,**d**) samples 3D printed using PJM technology: three-dimensional axonometric images.

**Figure 7:**
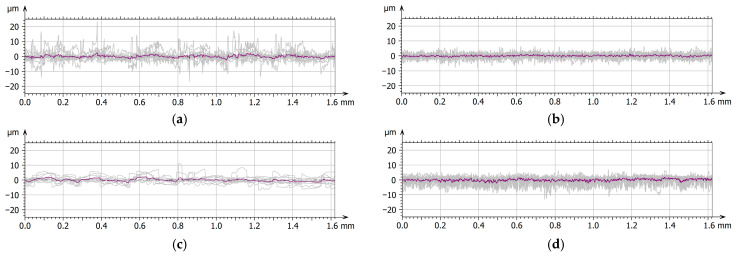
Surface texture: primary profiles with the medium profile marked—MED610 unpolishing (**a**), MED610 polishing (**b**), Ti–MED610 unpolishing (**c**), Ti–MED610 polishing (**d**).

Figure 6 presents the 3D axonometric images of the MED610 samples in both the unpolished and polished states, with and without the Ti coating. Figure 7 shows the primary surface profiles. The results of these analyses allow for an assessment of the influence of the polishing process and coating deposition on the microstructure and surface roughness in comparison with the base material. Table 3 summarises the amplitude parameters for all analysed surfaces, providing a quantitative assessment of topographical changes resulting from polishing and coating deposition.

On the surface of the unpolished, uncoated sample, characteristic droplets and irregularities typical of elements produced using PJM technology are visible, resulting from the deposition of successive polymer layers. The same droplet-like structures were also observed on the surface of the sample coated with the Ti layer; however, they were partially flattened—their height was approximately 20% lower compared with the uncoated surface. This phenomenon is likely due to the deposition of the coating, which fills micro-depressions and partially reduces height differences between individual layers of the polymer material. This observation is further supported by the amplitude parameters listed in Table 2. For the Sa (arithmetical mean height), Sq (root mean square height), and Sv (maximum pit depth) parameters, the differences between the unpolished coated and uncoated surfaces were approximately 20%. In contrast, for the Sp parameter (maximum peak height), a difference of up to 45% was observed. This result confirms that the deposited coating significantly smooths the highest points of the topography of the unpolished sample surface, reducing their height relative to uncoated areas. In the case of samples coated with the titanium layer, the effect of polishing was less pronounced, yet still noticeable. The values of the Sa and Sq parameters decreased by approximately 15% and 20%, respectively, whereas Sv and Sp increased by about 12% and 18%. The obtained results suggest that the presence of the Ti coating modifies the polishing process, limiting its effectiveness in reducing surface irregularities. The surface topography results further indicate that unpolished surfaces, due to their higher roughness and the presence of numerous micro-irregularities, may exhibit lower wear resistance under frictional conditions. These irregularities may promote local stress concentrations and intensify abrasive wear processes. From a biomedical standpoint, this is of particular importance—comparative studies of materials used in orthodontic appliances have shown that surfaces with lower roughness and higher smoothness display reduced tendencies for saliva, deposits, and bacterial accumulation, and therefore for biofilm formation. This is confirmed by the findings of Jeon et al. [35] as well as Ahn et al. [36], who demonstrated that surface roughness promotes strong bacterial adhesion.

### 3.3. Wettability and Adhesion

**Figure 8:**
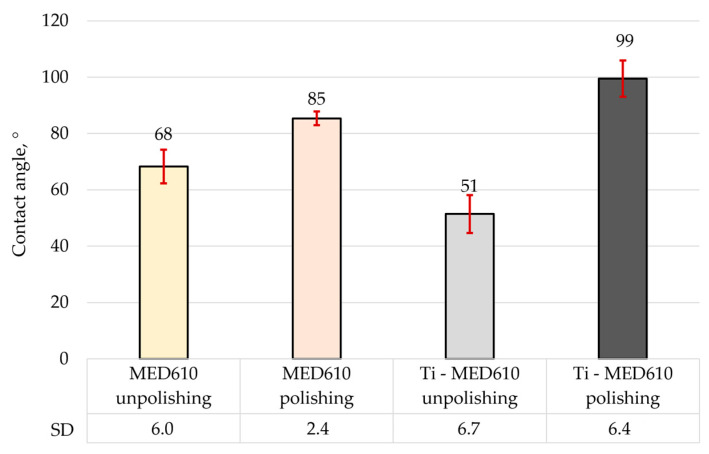
Average contact angle of MED610 material and after PVD of Ti coating.

**Figure 9:**
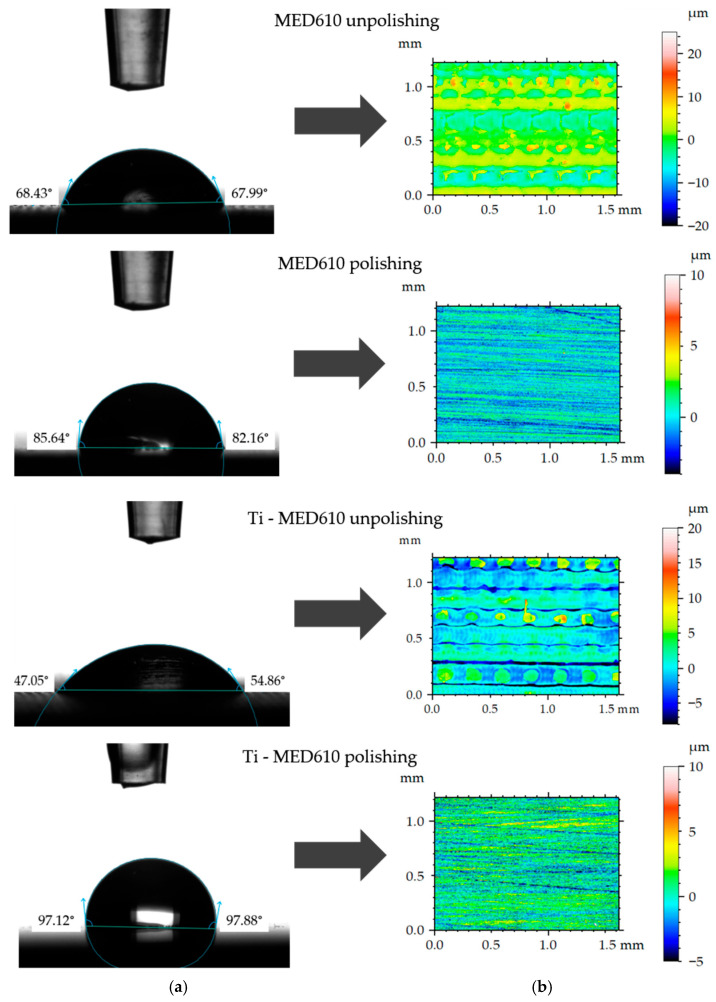
Representative contact angle images (**a**) and corresponding 2-D topographical images (**b**).

Figure 8 presents the mean contact angle values with error bars—standard deviation (SD) for the MED610 surface, calculated from ten measurements. Figure 9a shows images of artificial saliva droplets placed on the surface, while the corresponding 2D surface topography maps are shown in Figure 9b.

The wettability analysis revealed a clear influence of both the polishing process and the presence of the titanium coating on the contact angle values. The unpolished samples exhibited relatively low contact angles, indicating a hydrophilic character of these surfaces. The lowest contact angle was recorded for the unpolished MED610 sample with the Ti coating, measuring 51°, whereas the unpolished uncoated MED610 sample showed a value of 60°. This indicates that the presence of the titanium coating imparts a hydrophilic character to unpolished samples. For the polished surfaces, the contact angle values increased significantly—by approximately 20% for the MED610 sample and by as much as 50% for the Ti-coated MED610 sample (Ti-MED610), compared with the unpolished specimens. This demonstrates a shift in surface character from hydrophilic to hydrophobic.

The literature indicates that surface roughness affects not only wettability but also bacterial adhesion. Jeon et al. [35] emphasised that rough surfaces are more wettable and promote bacterial colonisation, whereas smoother surfaces exhibit lower wettability and reduced bacterial adhesion. Similar observations were reported by Martínez Gil-Ortega et al. [37], who noted that hydrophilic surfaces with contact angles around 90° favour a high level of bacterial colonisation. On the other hand, superhydrophilic surfaces, which rapidly adsorb water, and superhydrophobic surfaces, which are surrounded by an air layer, limit bacterial contact with the material, as confirmed by Oliveira et al. [38]. In the context of our results, this suggests that polishing and titanium coating can modulate the surface character and potentially influence bacterial adhesion, which is important in designing biomedical materials with controlled microbial colonisation. Studies from other research centres [35,37,38] show that surfaces with moderate hydrophilicity, characterised by contact angles in the range of approximately 60–80°, exhibit the most favourable biological properties. Such surfaces hinder the permanent adsorption of proteins and bacteria—processes that occur more rapidly on highly hydrophilic surfaces—while simultaneously reducing excessive deposition, which is typical of strongly hydrophobic surfaces. Additionally, moderately hydrophilic surfaces facilitate uniform spreading of saliva, supporting natural surface cleaning and limiting biofilm formation in the oral environment.

The images of the measurement liquid droplets shown in Figure 9 indicate that, on the unpolished surfaces, the artificial saliva droplet spread more rapidly, which reflects stronger liquid–substrate interactions and a hydrophilic character of the surface. The micro-irregularities remaining after printing promoted liquid retention by increasing the number of contact points between the droplet and the material. In the case of the polished surfaces, the droplet maintained a more spherical shape, indicating reduced liquid adhesion to the substrate and limited spreading.

This is confirmed by Rupp et al. [39], who observed that hydrophilic surfaces can facilitate initial interactions between the surface and the wetting liquid, which is important for wound healing and osseointegration. Furthermore, surface roughness has a significant impact on wettability and also plays an important role in the osseointegration process. Similarly, Liber-Kneć et al. [40] concluded that a hydrophobic surface character may increase bacterial adhesion.

**Figure 10:**
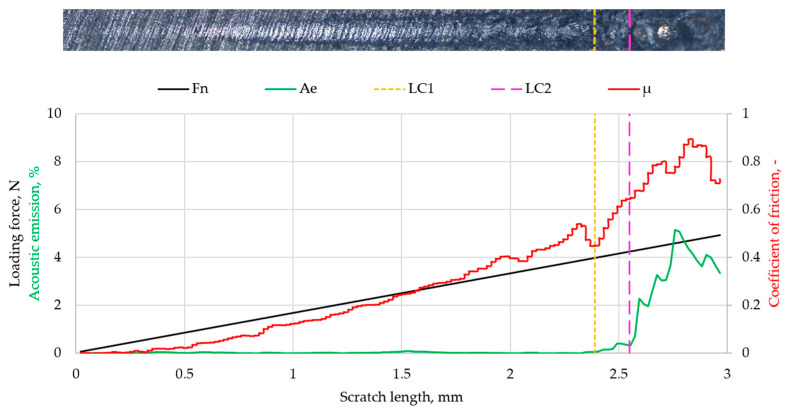
Scratch test results for Ti coatings.

Figure 10 shows the results of the scratch test performed on Ti coatings deposited on MED610. The figure presents optical images of the scratches, along with plots of the applied loading force (Fn), acoustic emission (Ae), friction coefficient (µ), and the critical force values (LC1 and LC2).

The results of the scratch test on the Ti coating deposited on MED610 indicated that the applied layer exhibited good adhesion to the substrate. A sudden increase in acoustic emission and friction coefficient was observed at a critical load of approximately 3.92 N (LC1). Since no spalling of the deposited layer was observed in the optical images, this jump is attributed to the surface roughness of the Ti–MED610 system. Increasing the load to 4.19 N (LC2) caused another jump in both parameters and led to delamination of the coating. For thin coatings (with thickness on the order of nanometres), the values of the critical forces LC1 and LC2 in this range indicate good adhesion of the coating to the substrate. This interpretation is consistent with the approach presented by Valli [41], who emphasised that LC1 typically corresponds to the initiation of cohesive damage within the coating, whereas LC2 is the critical adhesion parameter that marks the onset of coating detachment from the substrate. According to this framework, the LC1 and LC2 values obtained for the examined system—characteristic of thin coatings with nanometre-scale thickness—indicate good interfacial bonding between the Ti coating and the MED610 substrate.

### 3.4. Tribological Test and Wear Analysis of Samples After Testing

**Figure 11:**
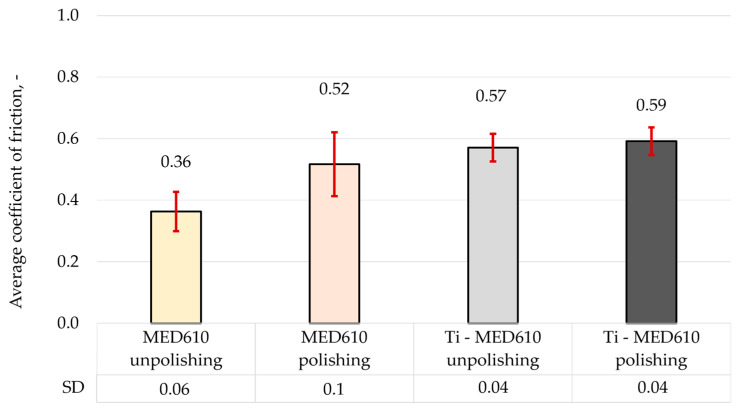
Average coefficient of friction of MED610 material and after PVD of Ti coating.

**Figure 12:**
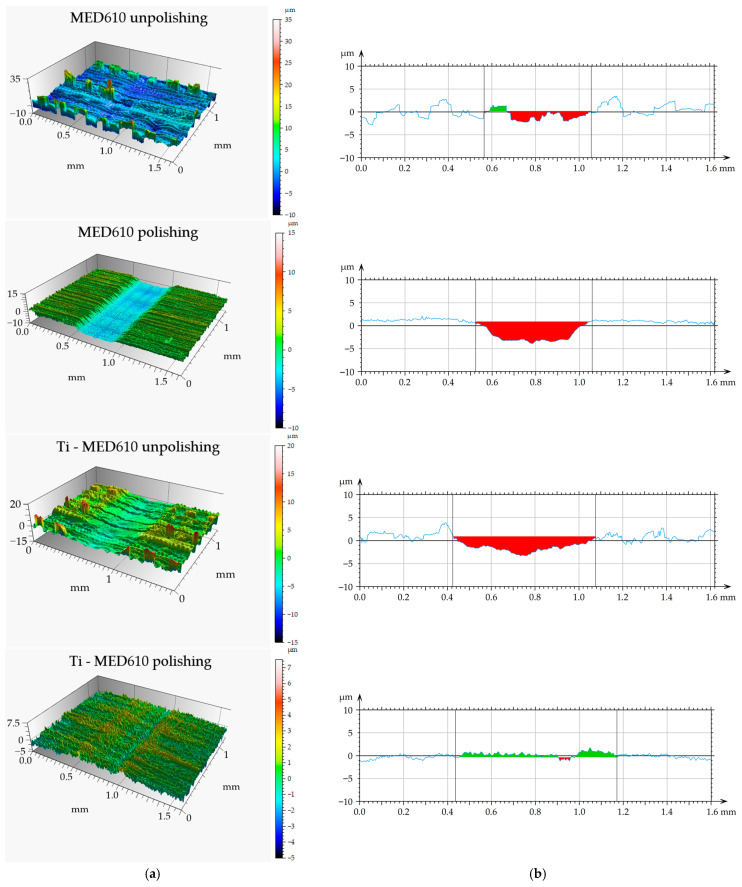
Views of wear track (**a**) and primary profiles on the cross-section (**b**) of MED610 material and after PVD of Ti coating.

**Figure 13:**
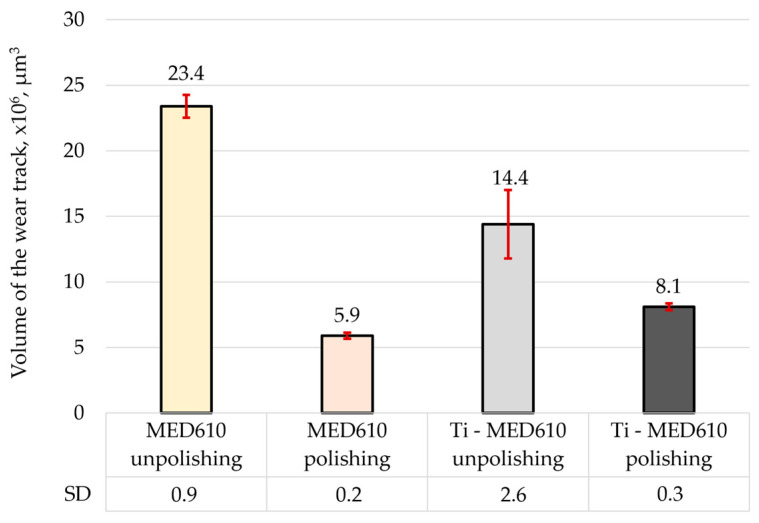
Volume of the wear track for the tested samples.

Figure 11 presents the mean friction coefficient values with error bars—standard deviation (SD) for the MED610 samples without the coating and those with the titanium (Ti) coating, while Figure 12 shows the three-dimensional axonometric views and primary profiles of the wear tracks, wherebuild-ups are indicated in green, whereas indentations are indicated in red. The use of a 10 mm amplitude and confocal microscopy enabled measurement of the entire wear area, allowing for a detailed analysis of the wear track and precise determination of the wear track volume (Figure 13). All values were calculated based on three independent measurement series.

The results of the friction coefficient measurements indicate that the polishing process contributes to an increase in its value by approximately 31% for the uncoated MED610 material. This phenomenon can be attributed to the smoothing of micro-irregularities, which reduces the surface’s ability to retain the lubricant (artificial saliva). Additionally, it was observed that the application of the titanium (Ti) coating to both unpolished and polished samples results in a further increase in the friction coefficient—by approximately 36% and 12%, respectively, compared with the base MED610 material. The friction coefficient values for the Ti-coated samples were similar (0.57 and 0.59), regardless of surface topography, suggesting that the presence of the coating stabilises the tribological behaviour of the material by reducing the influence of substrate roughness on the friction process. In the case of the polished samples, the increase in the friction coefficient after the coating deposition was associated with a rise in local surface roughness. This correlation between topography and friction value is also confirmed by Bartosova et al. [20], who demonstrated that surface bombardment increased the roughness of the samples, and consequently, their friction coefficient. According to Grzesik [42], in addition to the Sa and Sq parameters, Sku and Ssk also have a significant impact on friction and wear. A negative Ssk value contributes to an increase in the friction coefficient. This was confirmed for both polished and unpolished samples, as the deposition of the coating resulted in a negative Ssk value (Table 3), leading to higher average friction coefficient values.

The results of the volumetric wear analysis indicate that the fastest-wearing material in the friction pair with the steel ball was the unpolished and uncoated MED610 sample. It was observed that the polishing process significantly reduced the volumetric wear of the material—by approximately 75% for the uncoated samples and by 44% for the samples with the Ti coating. This effect can be attributed to the reduction in surface micro-irregularities (peaks), which limits local micro-impacts and decreases the intensity of abrasive wear. The application of the titanium (Ti) coating to the unpolished sample reduced the volumetric wear by approximately 40% compared with the uncoated surface. In contrast, for the polished sample, the coating resulted in a slight increase in wear—by around 27%. This indicates that the protective effectiveness of the Ti coating is strongly dependent on the substrate surface topography, and that its adhesion and ability to distribute loads uniformly may be limited on smoother surfaces. The obtained results demonstrate the crucial role of surface roughness in shaping the tribological properties of polymer materials. Moderate roughness promotes the retention of the lubricant (artificial saliva), which may reduce friction but simultaneously increase the intensity of abrasive wear. On the other hand, excessive smoothing of the surface reduces lubrication effectiveness while also decreasing the volumetric material loss. Furthermore, Al-Samarai et al. [43], Bayer et al. [44], and Barrett et al. [45] observed in their studies that the mass- and volume-specific wear rate decreases with a reduction in surface roughness, which was also confirmed in the present study.

Additionally, a statistical analysis was conducted to determine the probability of the influence of individual technological factors on the output parameters of the study.The effect of surface polishing and Ti coating on wettability, friction and wear of 3-D-printed samples is summarised in Table 4. In this table, a single asterisk indicates a lower probability of the outcome, while three asterisks represent the highest probability.

A detailed analysis of the experimental results indicated that polishing leads to an increase in both the contact angle and the coefficient of friction, while it simultaneously reduces volumetric wear. Conversely, unpolished samples exhibited the opposite behaviour.

For samples coated with Ti, the most pronounced differences were observed in terms of wear. Unpolished Ti-coated samples demonstrated approximately 38% lower wear compared to their uncoated counterparts. In contrast, when these samples were polished, the wear increased by around 37%. These findings suggest that surface preparation significantly influences wear behaviour, with unpolished surfaces exhibiting reduced material loss.

## 4. Conclusions

Based on the experimental analyses performed, the following conclusions can be drawn regarding the effects of polishing and Ti-coating deposition on the surface and tribological properties of the MED610 material:

1. The PVD process enabled the deposition of a uniform ~300 nm titanium coating with good adhesion to the MED610 substrate.
2. Surface topography measurements showed that the Ti coating reduced the characteristic roughness of PJM-manufactured components, lowering amplitude parameters by 20–45%.
3. Wettability tests demonstrated that unpolished samples exhibited lower contact angles, while polishing increased the contact angle and shifted the surface character from hydrophilic to hydrophobic.
4. Tribological measurements revealed that polishing increased the friction coefficient of MED610 by approximately 31%, likely due to reduced lubricant retention, while Ti-coated samples exhibited similar friction values (~0.57–0.59), indicating stabilisation of frictional behaviour by the coating. Wear analyses showed that polishing significantly decreased volumetric wear (by ~75% for uncoated and ~44% for Ti-coated samples). Ti deposition reduced wear by ~40% on unpolished surfaces, whereas on polished surfaces it caused a slight wear increase of about 27%.
5. Overall, the results confirm that both surface roughness and the titanium coating substantially affect the tribological behaviour and wettability of MED610. Improved wear resistance and favourable wettability in artificial saliva highlight the potential of this surface modification for medical devices intended for contact with mucous membranes, and provide a basis for further studies on enhancing the biocompatibility and durability of 3D-printed polymer materials.

## Figures and Tables

**Table 1:** Chemical composition of the lubricant—artificial saliva [28].

Artifical Saliva, g/dm^3^					
NaCl	KCl	CaCl_2_ ∗ 2H_2_O	NaH_2_PO_4_ ∗ 2H_2_O	Na_2_S ∗ 9H_2_O	Urea
0.4	0.4	0.795	0.780	0.005	1.0

**Table 2:** Coating deposition parameters.

Cleaning			Coating		
Ar, sccm	Time, min	RF, W	Ar, sccm	Time, min	DC, W
30	20	100	30	180	100

**Table 3:** Amplitude parameters for representative samples.

	Sa	Sq	Sv	Sp	Ssk	Sku
	µm	µm	µm	µm	-	
MED610 unpolished	2.8	3.5	31.1	28.3	0.002	3.3
MED610 polished	1.2	1.6	11.6	16.0	−0.24	4.0
Ti-MED610 unpolished	2.0	2.9	23.1	15.3	−0.4	4.8
Ti-MED610 polished	1.7	2.3	26.2	18.5	−1.0	4.5

**Table 4:** Effect of surface polishing and Ti coating on wettability, friction and wear of 3-D-printed samples (mean ± standard deviation).

Surface Condition	Coating	Avg. Contact Angle (°)	Avg. Coefficient of Friction	Volume of Wear Track (×10^6^)
Unpolished	Without Ti	68 ± 6	0.36 ± 0.06	23.4 ± 0.9
Polished		85 ± 2.4 **	0.52 ± 0.1 *	5.9 ± 0.2 ***
Unpolished	With Ti	51 ± 6.7 *	0.57 ± 0.04 **	14.4 ± 2.6 **
Polished		99 ± 6.4 **	0.59 ± 0.04 **	8.1 ± 0.3 ***

Statistical significance compared to the unpolished, uncoated sample: *—*p* < 0.05, **—*p* < 0.01, ***—*p* < 0.001.

## Data Availability

The original contributions presented in the study are included in the article. Further inquiries can be directed to the corresponding author.
